# Heat exposure and hospitalizations for chronic kidney disease in China: a nationwide time series study in 261 major Chinese cities

**DOI:** 10.1186/s40779-023-00478-4

**Published:** 2023-09-05

**Authors:** Fu-Lin Wang, Wan-Zhou Wang, Fei-Fei Zhang, Su-Yuan Peng, Huai-Yu Wang, Rui Chen, Jin-Wei Wang, Peng-Fei Li, Yang Wang, Ming-Hui Zhao, Chao Yang, Lu-Xia Zhang

**Affiliations:** 1 Institute of Medical Technology, Peking University Health Science Center, Beijing, 100191 China; 2 National Institute of Health Data Science at Peking University, Beijing, 100191 China; 3 Department of Occupational and Environmental Health Sciences, School of Public Health, Peking University, Beijing, 100191 China; 4 Renal Division, Department of Medicine, Peking University First Hospital, Peking University Institute of Nephrology, Beijing, 100034 China; 5 Research Units of Diagnosis and Treatment of Immune-Mediated Kidney Diseases, Chinese Academy of Medical Sciences, Beijing, 100034 China; 6 Advanced Institute of Information Technology, Peking University, Hangzhou, 311215 China; 7 National Climate Center, China Meteorological Administration, Beijing, 100081 China; 8 Peking-Tsinghua Center for Life Sciences, Beijing, 100034 China

**Keywords:** Chronic kidney disease, Hospitalization, Climate change, Temperature, Time-series study

## Abstract

**Background:**

Climate change profoundly shapes the population health at the global scale. However, there was still insufficient and inconsistent evidence for the association between heat exposure and chronic kidney disease (CKD).

**Methods:**

In the present study, we studied the association of heat exposure with hospitalizations for cause-specific CKD using a national inpatient database in China during the study period of hot season from 2015 to 2018. Standard time-series regression models and random-effects meta-analysis were developed to estimate the city-specific and national averaged associations at a 7 lag-day span, respectively.

**Results:**

A total of 768,129 hospitalizations for CKD was recorded during the study period. The results showed that higher temperature was associated with elevated risk of hospitalizations for CKD, especially in sub-tropical cities. With a 1 °C increase in daily mean temperature, the cumulative relative risks (*RR*) over lag 0–7 d were 1.008 [95% confidence interval (CI) 1.003–1.012] for nationwide. The attributable fraction of CKD hospitalizations due to high temperatures was 5.50%. Stronger associations were observed among younger patients and those with obstructive nephropathy. Our study also found that exposure to heatwaves was associated with added risk of hospitalizations for CKD compared to non-heatwave days (*RR* = 1.116, 95% CI 1.069–1.166) above the effect of daily mean temperature.

**Conclusions:**

Short-term heat exposure may increase the risk of hospitalization for CKD. Our findings provide insights into the health effects of climate change and suggest the necessity of guided protection strategies against the adverse effects of high temperatures.

**Supplementary Information:**

The online version contains supplementary material available at 10.1186/s40779-023-00478-4.

## Background

Climate change poses a huge threat to human health [1]. Increasing epidemiological evidence has linked climate change, particularly ambient temperature, to the global disease burden [2–6]. A multi-country study based on 384 locations estimated that about 7.71% of mortality between 1985 and 2012 is attributable to non-optimal ambient temperature over a short-term exposure of 21 d of lag [6]. High temperature-related mortality and disease morbidity are expected to increase from 2010 to 2050 [7, 8].

High temperature exposure may lead to heat stress responses (e.g., dehydration), induce changes in renal vascular hemorheology, and contribute to the onset and progression of kidney diseases [9–11]. Chronic kidney disease (CKD) has been acknowledged as a global public health issue [9]. Importantly, about 697.5 million people suffered from CKD in 2017, contributing to 1.2 million deaths worldwide [12]. The global disease burden attributable to CKD is still rapidly ascending, particularly in developing countries [12]. According to a national representative survey, the overall prevalence of CKD among Chinese adults is estimated to be 10.8% [13], and is higher than the global prevalence of 9.1% [12]. Given the increasing extreme heat events and the challenging burden of CKD, assessing the effect of heat exposure on the development of CKD quantitatively has profound implications. Understanding the current heat-related burden for CKD and identifying the most vulnerable sub-populations can help to inform early public health interventions during hot season, and then reduce the kidney damage from heat exposure. Such findings could also provide evidence for the heat warning system design and patients’ self-management in hot weather.

Several epidemiological studies have proved the consistent and positive association between heat exposure and increased risk of kidney-related morbidity and mortality, such as acute kidney injury, urolithiasis, and urinary tract infections [9, 11, 14–17]. However, unlike those diseases confined to kidneys, our previous study indicated that CKD is characterized by multiple etiologies with different systematic pathophysiological process [18, 19]. The spectrum of CKD is evolving and varies greatly among different regions [18, 20]. Therefore, there is a necessity to assess the effect of global warming on CKD in different countries. Previous studies have found apparently inconsistent results in different regions on this topic. For instance, high temperature has been linked to increased risks of hospitalizations for CKD during warm season in Vietnam [14] and CKD-related emergency room visits in South Australia and Taiwan [16, 21], but not to hospitalizations for CKD in Brazil [9] and CKD-related hospital admissions through the emergency department in South Korea [11]. There are still several knowledge gaps regarding the adverse effects of high temperatures on CKD, due to the lack of studies that 1) examined the effect on specific etiologies of CKD, especially in the context of the changing spectrum of CKD in China; 2) evaluated the attributable fraction (AF) due to high temperatures for CKD hospitalizations; and 3) assessed the effect of both high temperature and consecutive extreme heat.

In this nationwide multi-city study, using data on CKD hospitalization cases during 2015–2018 in 261 major cities and complex identification strategies for cause-specific CKD, we aimed to systematically evaluate the association between short-term heat exposure and hospitalizations for CKD as well as its major etiologies in China, and whether the association varied across different subpopulations. Under the background of global warming, this study provided insights into the heat-related disease burden of CKD among specific etiologies and population characteristics, which has important implications for tailoring appropriate prevention strategies against the adverse effects of heat exposure, especially for the vulnerable population.

## Methods

### Hospitalization data collection

Daily hospitalization data from January 1, 2015 to December 31, 2018 was retrieved from the Hospital Quality Monitoring System (HQMS), a patient-level national database for hospital accreditation in China. Since 2013, all tertiary hospitals in China have been requested to submit electronic inpatient discharge records to the HQMS daily. As of December 2018, HQMS has covered more than 75% of tertiary hospitals in 31 provinces, autonomous regions, and municipalities directly under the central government (excluding Hong Kong, Macao, and Taiwan). A more detailed description of the HQMS database can be found in previous studies [18, 19, 22, 23].

We identified adult inpatients (≥ 18 years) with a principal discharge diagnosis of CKD through the International Classification of Diseases-10 (ICD-10) codes, which are described in previous publications (Additional file 1: Table S1) [18, 19, 24]. According to our previous study, CKD is characterized by multiple etiologies including diabetic kidney disease (DKD), hypertensive nephropathy (HTN), glomerulonephritis (GN), renal tubulointerstitial diseases, obstructive nephropathy (ON), and CKD due to other reasons [18, 19]. Since the number of hospitalizations for renal tubulointerstitial diseases was relatively small, we only focused on the four majority etiologies: DKD, HTN, GN and ON. For hospitalizations with multiple CKD causes, the etiology with higher rankings of diagnosis would be selected. The specificity of CKD identification by ICD-10 coding in the HQMS has been reported to be 97.8% [25]. We also extracted the permanent residence, age, sex, and admission date of each patient for our analysis. Daily number of hospitalizations for CKD were calculated according to the admission date of each patient. As the detailed residence addresses were unavailable due to the administrative protection of personal privacy, we conducted a time series analysis at the city level instead of the individual level. This widely used approach can be used to evaluate associations between exposure and health at the population level on a daily basis [3, 6].

**Fig. 1:**
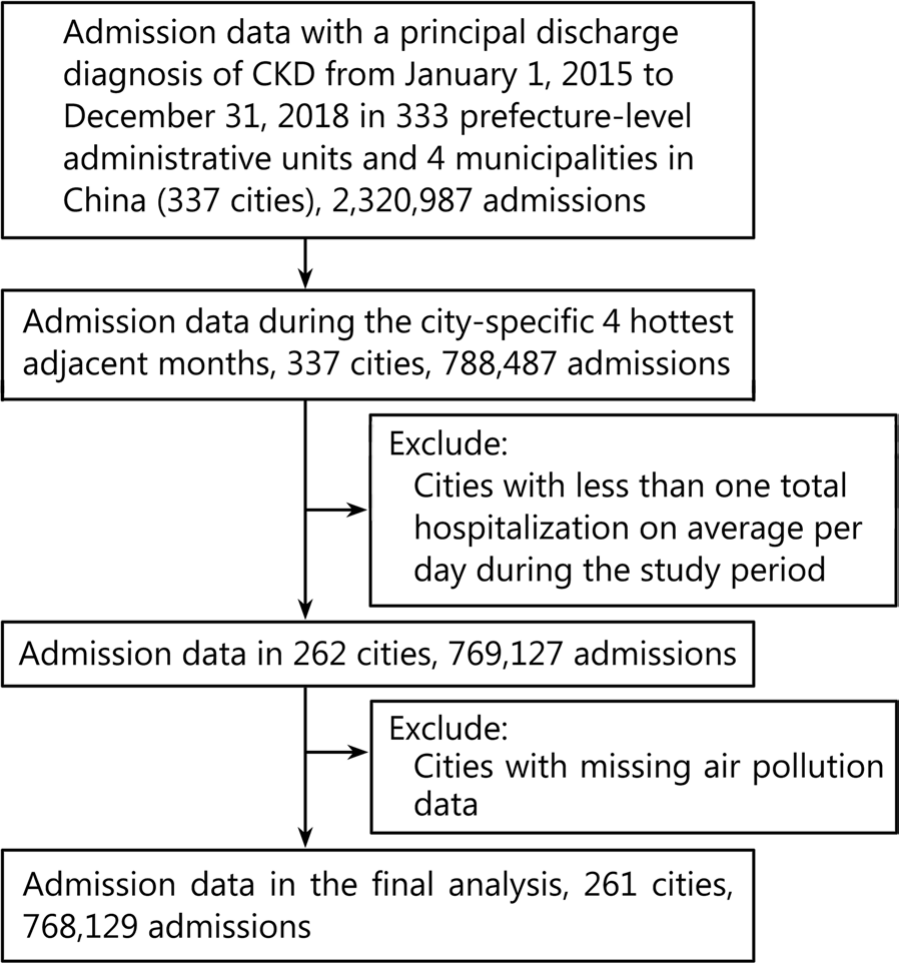
Flow chart of study cities selection. CKD chronic kidney disease

We obtained the daily CKD admission data from 2015 to 2018 in 333 prefecture-level administrative units and 4 municipalities in China, and we excluded cities missing air pollution data or cities with less than one total hospitalization on average per day during the study period to ensure sufficient statistical power when fitting time series data [26]. Finally, hospitalization data of 261 cities (Fig. 1) in China was included in our analyses, which accounted for more than 77% of the total number of prefecture-level cities in China and covered a population of more than 1.2 billion people. These cities cover five temperature zones in China, with most of them in temperate, warm-temperate and sub-tropical zones and a few in tropics and the Tibetan Plateau zones. This study was approved by the Ethics Committee of Peking University First Hospital (2021–020), and informed consent was waived by its Ethics Committee.

### Definition of heat exposure

We first investigated the health risk of high temperature, which is measured by daily mean temperature in hot season. We only included time-series data of hot seasons because the focus of our study was heat-related CKD hospitalizations [16]. Hot season was defined as the four hottest adjacent months for each city [27]. Additionally, according to previous studies, the association between heat exposure and risk of health outcomes may include the independent health risk of daily high temperature and the added health risk attributed to the duration of extreme heat [27, 28]. Except for ambient high temperature, we further studied whether heatwaves had added effects on CKD hospitalizations above the effect of daily mean temperature. Heatwave was defined as ≥ 95th percentile of city-specific distribution of daily mean temperature during the hot season and lasted for three or more consecutive days [29].

### Environmental data collection

We used daily mean temperature to characterize the ambient temperature exposure in this study. Potential confounders, such as daily mean relative humidity and daily mean air pollution concentration were also included. We derived daily meteorological data for each city from January 1, 2015 to December 31, 2018 from the China Meteorological Data Sharing Service System (https://data.cma.cn/). Daily air pollution data was obtained from China’s National Urban Air Quality Real-Time Publishing Platform (https://air.cnemc.cn:18007/). Then time series data were generated by merging daily hospitalization data, daily meteorological data, and daily air pollution data by date and city name.

### Statistical analysis

A two-stage method was used to examine the national pooled association between ambient temperature and hospitalizations for CKD [3, 30, 31]. In the first stage, a standard time series regression model [3, 32] was applied to estimate city-level relationships. To account for the potential delayed effects of ambient temperature, a cross-basis function was generated through the distributed lag non-linear model [33, 34]. The final model structure has the following formula:

1 $$\begin{aligned} Log\left[ {E\left( Y \right)} \right] & = cb\left( {temp} \right) + cb\left( {humidity} \right) + PM_{10} + O_{3} \\ & \quad + ns\left( {calendar\,day} \right) + ns\left( {DOY} \right) + DOW + PH \\ \end{aligned}$$

where $E\left( Y \right)$ is the estimated daily hospitalizations on a certain day. $cb\left( {Ttemp} \right)$ is the cross-basis function of ambient temperature, which describes the temperature-lag-response relationship. For the temperature-response dimension, Generalized Cross Validation (GCV) was used to test the performance of the nonlinear model [natural cubic spline function with 3 degrees of freedom (df) and equally spaced knots] and the linear model [35]. The linear model had a lower GCV scores (sum of 261 cities, 354.59) than the nonlinear model (359.19, Additional file 1: Fig. S1), and therefore the final cross-basis function of temperature included a linear function in the temperature-response dimension and a natural cubic spline function for the lag-response dimension, which had 3 dfs with equally spaced knots [9]. We set maximum lag in the cross basis function as 7 d to characterize the potential delayed effects [9]. $cb\left( {humidity} \right)$ represents the cross-basis function of daily mean relative humidity for lag 0–7 d with the 3 dfs natural cubic splines in humidity and lag dimension respectively. As illustrated in previous studies [36–39], air pollution may affect the prevalence of kidney diseases. Therefore, we adjusted air pollution factors in our model with the same lags as the high temperature, which were calculated by the moving averages. Two air pollutants including particles with an aerodynamic diameter of 10 µm or less and ozone were considered. $ns()$ presents the natural cubic spline for non-linear variables. Consistent with previous studies, we introduced the calendar day (with 3 dfs per year) and the day of year (DOY, with 3 dfs) variables to control the long time and seasonal trend [33]. A dummy variable for the day of the week and a dichotomous variable for a public holiday were also introduced [35, 40]. GCV values were used to choose the appropriate dfs for the spline functions.

In the second stage of our method, a random-effects meta-analysis without predictors was used to pool city-specific estimates at the national level. The best linear unbiased prediction of city-specific cumulative associations between temperature and hospitalizations for CKD were also obtained in the second stage, using the fitted meta-analysis model [41, 42]. The best linear unbiased prediction method provided more precise city-specific estimations by a trade-off between city-specific association in the first stage and the pooled association in the second stage, which would be further used in the calculation of AF [3, 41]. For each city, we calculated the daily relative risk (*RR*) compared to the city-specific minimum daily mean temperature during the study period. The attributable case (AC) on a specific day $i$ was estimated using $AC_{i} = N_{i} \times \left( {RR_{i} - 1} \right)/RR_{i}$, where $N_{i}$ was the city-specific average number of hospitalizations from day $i$ to day $i + 7$.  The total AC and 95% confidence interval (CI) were generated by summing the AC (95% CI) of all cities, and the total AF (95% CI) was then calculated through dividing the total AC (95% CI) by the total number of hospitalizations for CKD during the study period [9, 43].

To explore the association between temperature and hospitalizations for CKD across populations with different characteristics, we stratified the data according to three major temperature zones in China (temperate zone, warm-temperate zone and sub-tropical zone), the etiologies of CKD, sex, and age group (18–40, 40–60, ≥ 60 years old). The same model in formula (1) with a linear function for ambient temperature was used in the stratified analysis. We tested the statistical significance of differences between subgroup estimates using a previously established approach [44]. To be specific, difference between log *RR* could be tested based on the estimates (*E*_1_ and *E*_2_) and standard errors (*SE*_1_ and *SE*_2_): $Z = \left( {E_{1} - E_{2} } \right)/\sqrt {\left( {SE_{1}^{2} + SE_{2}^{2} } \right)}$.

Besides daily mean temperature, the added health risk of heatwave days compared with non-heatwave days was evaluated by adding another cross-basis function to formula (1), which combined a linear function for an indicator variable of heatwave days and a 3 dfs natural cubic spline function for lag 0–7 d. Then the same two-stage method as mentioned above was applied.

In the sensitivity analysis, we applied different lag spans and df for spline functions of confounders (relative humidity, calendar day, and DOY) to examine the robustness of our results. We also tested whether the exposure–response curve was sensitive to the knot placements in cross-basis function. In addition, since other air pollutants such as particles with an aerodynamic diameter of 2.5 µm or less and nitrogen dioxide might also have effects on kidney health, we adjusted different air pollutants to evaluate whether our results remained robust. All statistical analyses were performed with R software (version 3.6.2), R packages including ‘mgcv’, [45] ‘dlnm’, [33] and ‘mvmeta’ were used [42]. We presented results as estimated *RR* with a 95% CI of hospitalizations for CKD with every 1 °C increase in daily mean temperature. Statistical significance was defined as a two-sided *P*-value of < 0.05.

## Results

Table 1 summarizes the number of hospitalizations related to CKD grouped by three major temperature zones in China, and other individual characteristics including age group, sex, and CKD etiologies. During the study period of hot season from 2015 to 2018, a total of 768,129 hospitalizations with a principal discharge diagnosis of CKD were documented in the HQMS database. It was observed that patients of 40–60 age group (41.08%), male (57.06%) and patients with GN (25.09%) accounted for a higher proportion of all hospitalizations. The average daily hospitalization number and ambient temperature during the study period were (6.02 ± 7.81) and (24.33 ± 3.74) °C, respectively. The sub-tropical zone had both highest daily hospitalization number (7.09 ± 8.74) and mean temperature (25.46 ± 3.06) °C among all three zones. The 95th percentile of nationwide daily mean temperature was 29.64 °C.

**Table 1:** Summary of hospitalizations for CKD and daily mean temperature in 261 Chinese cities during the hot season from 2015 to 2018, grouped by three major temperature zones

Variable	Overall	Temperate zone	Warm-temperate zone	Sub-tropical zone^a^
Total [*n* (%)]	768,129 (100.00)	53,065 (6.91)	174,080 (22.66)	526,363 (68.53)
Age group [*n* (%)]				
18–40 years	160,086 (20.84)	10,222 (19.26)	39,526 (22.71)	107,308 (20.39)
40–60 years	315,574 (41.08)	21,810 (41.10)	69,788 (40.09)	217,707 (41.36)
≥ 60 years	292,469 (38.08)	21,033 (39.64)	64,766 (37.20)	201,348 (38.25)
Sex [*n* (%)]				
Male	438,318 (57.06)	29,502 (55.60)	99,021 (56.88)	301,281 (57.24)
Female	329,811 (42.94)	23,563 (44.40)	75,059 (43.12)	225,082 (42.76)
Etiology [*n* (%)]^b^				
Diabetic kidney disease	104,893 (13.66)	9183 (17.31)	28,447 (16.34)	64,958 (12.34)
Hypertensive nephropathy	96,345 (12.54)	7431 (14.00)	19,496 (11.20)	68,256 (12.97)
Glomerulonephritis	192,720 (25.09)	16,057 (30.26)	55,657 (31.97)	117,531 (22.33)
Obstructive nephropathy	165,212 (21.51)	4869 (9.18)	20,730 (11.91)	137,039 (26.04)
Daily hospitalizations (*n*, mean ± SD)	6.02 ± 7.81	3.28 ± 4.22	5.16 ± 6.69	7.09 ± 8.74
Daily mean temperature (°C, mean ± SD)	24.33 ± 3.74	20.66 ± 3.96	23.71 ± 3.51	25.46 ± 3.06
95th percentile of temperature (°C)	29.64	26.18	28.97	30.00

*CKD* chronic kidney disease, *SD* standard deviation

^a^There are also some cities in the tropics and the Tibetan Plateau zones, but only account for a very small proportion of the total CKD hospitalization. ^b^Four majority etiologies of CKD are listed, and there are also some hospitalizations for CKD due to other etiologies

**Fig. 2:**
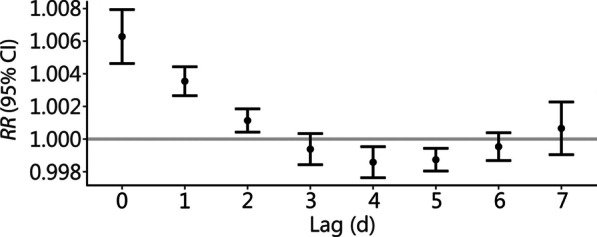
Association between daily mean temperature and hospitalizations for CKD at lag 0–7 d. CKD chronic kidney disease, RR relative risk

The increase in daily mean temperature in hot season was associated with an increased risk of hospitalizations for CKD. With a 1 °C increase in daily mean temperature, the cumulative *RR* over lag 0–7 d were 1.008 (95% CI 1.003–1.012) for nationwide, 1.011 (95% CI 1.006–1.017) for cities in the sub-tropical zone, while the effect was insignificant for cities in the temperate and warm-temperate zone (Table 2). The AF due to high temperatures was 5.50% for overall CKD hospitalizations, and 7.12% for patients living in the sub-tropical zone. Figure 2 showed that the associations between high temperature and hospitalizations for CKD were strongest at the lag 0 d and followed by a hospitalization displacement (harvesting effect) after lag 3 d.

**Table 2:** Relative risk (*RR*) and attributable fraction of hospitalizations for CKD associated with ambient high temperature (every 1 °C increase in daily mean temperature in hot season), grouped by three temperature zones

Variable	*RR* (95% CI)	*P*-values for difference	Attributable fraction (%)
Overall	1.008 (1.003–1.012)	–	5.500 (− 13.549 to 20.730)
Temperature zone			
Temperate zone	0.999 (0.985–1.013)	Ref	–
Warm-temperate zone	1.004 (0.996–1.012)	0.544	–
Sub-tropical zone	1.011 (1.006–1.017)	0.120	7.124 (− 7.419 to 19.355)

*CKD* chronic kidney disease, *CI* confidence interval, *Ref* reference

**Fig. 3:**
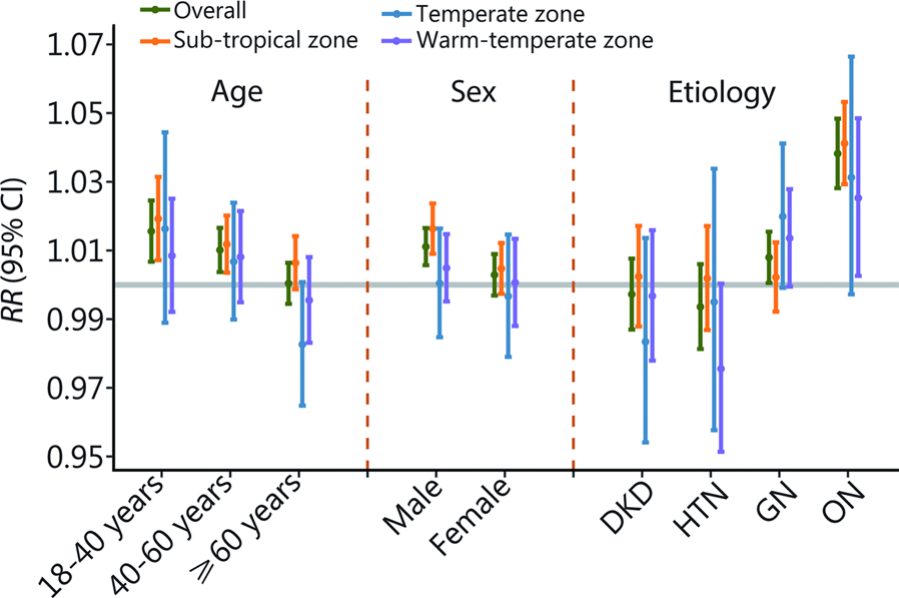
The cumulative association stratified by age, sex, and etiology of CKD. The cumulative association between ambient high temperature (every 1 °C increase in daily mean temperature in hot season) and the risk of hospitalizations for CKD over 0–7 d, stratified by age, sex and etiologies of CKD. The gray horizontal line indicates *RR* = 1. CKD chronic kidney disease, DKD diabetic kidney disease, HTN hypertensive nephropathy, GN glomerulonephritis, ON obstructive nephropathy, RR relative risk

The estimated associations of temperature with hospitalizations for CKD stratified by age group, sex, and etiologies of CKD were displayed in Fig. 3. Stronger associations were observed among younger patients aged 18–40 (*RR* = 1.016, 95% CI 1.007–1.025) and patients aged 40–60 (*RR* = 1.010, 95% CI 1.004–1.017) than patients over 60 years (*RR* = 1.000, 95% CI 0.994–1.006) with a significant difference (*P* < 0.05). Higher association was also found in male (*RR* = 1.011, 95% CI 1.006–1.017) than female (*RR* = 1.003, 95% CI 0.997–1.009), though the difference was not statistically significant (*P* = 0.054). Additionally, when stratified hospitalizations by etiologies of CKD, high temperature was associated with the hospitalizations for ON (*RR* = 1.038, 95% CI 1.028–1.048) and GN (*RR* = 1.008, 95% CI 1.001–1.015), while the association was insignificant for DKD and HTN hospitalizations. The lag structure curve showed that the associations between high temperature and hospitalizations for CKD was highest at the lag 0 d and followed by a hospitalization displacement among all the subgroups (Additional file 1: Fig. S2).

As shown in Table 3, exposure to heatwaves characterized by extreme hot and long durations was associated with added risk of hospitalizations for CKD compared to non-heatwave days above the effect of daily mean temperature (*RR* = 1.116, 95% CI 1.069–1.166). The added risk of heatwave existed in a wider population than the effect of independent high temperature, as it was significant in all the subgroups stratified by age group, sex, and etiologies of CKD.

**Table 3:** Added risk of hospitalization for CKD associated with heatwave days by three temperature zones, age, sex, and etiologies of CKD

Variable	*RR* (95% CI)	*P*-values for difference
Overall	1.116 (1.069–1.166)	–
Temperature zone		
Temperate zone	0.978 (0.828–1.155)	Ref
Warm-temperate zone	1.216 (1.092–1.355)	0.031
Subtropical zone	1.104 (1.050–1.161)	0.172
Age (years)		
18–40	1.228 (1.126–1.339)	Ref
40–60	1.184 (1.110–1.262)	0.507
≥ 60	1.082 (1.014–1.154)	0.022
Sex		
Male	1.157 (1.093–1.224)	Ref
Female	1.109 (1.044–1.178)	0.316
Etiology of CKD		
Diabetic kidney disease	1.170 (1.043–1.311)	Ref
Hypertensive nephropathy	1.187 (1.058–1.331)	0.861
Glomerulonephritis	1.170 (1.082–1.265)	> 0.999
Obstructive nephropathy	1.258 (1.158–1.367)	0.314

*CKD* chronic kidney disease, *CI* confidence interval, *Ref* reference, *RR* relative risk

We also estimated the effect of heat exposure on hospitalizations for CKD under different model settings, including maximum lag days, dfs (for spline functions of relative humidity, DOY, calendar day), adjusted air pollutants, and knot placements of temperature-response dimension in the cross-basis function. These results highlight the robustness of our findings (Additional file 1: Table S2 and Fig. S3).

## Discussion

This study systematically evaluated the associations between short-term heat exposure and risks of cause-specific CKD based on a national database that covers a wide range of geographical regions in China. We found that temperature increase was consistently positively associated with increased risks of hospitalizations for CKD in China, especially among cities in the sub-tropical zone. Stronger associations were observed among younger patients and patients with ON. Exposure to heatwaves was associated with added risk of hospitalizations for CKD above the effect of daily mean temperature.

Our study showed that increments in daily mean temperature were associated with increased risks of CKD during hot season in China. More than 5% of hospitalizations for CKD was estimated to be attributable to high temperatures, which indicated a profound health effect of global warming on CKD. The results were congruent with some previous studies focusing on the effects of temperature on kidney-related diseases. A study covering 85% of all Medicare enrollees in 1943 counties of the United States found that the risk of hospitalizations for kidney failure was higher on heatwave days relative to matched non-heatwave days (*RR* = 1.14, 95% CI 1.06–1.23) [46]. Meanwhile, the extremely high temperature was also found to increase the risk of emergency department visits for CKD [16, 21]. A study involving 1959 emergency department cases with CKD in South Australia showed that a 1 °C increase in daily minimum temperature was associated with a 1.7% increase in the risk of daily emergency department admissions for CKD (*RR* = 1.017, 95% CI 1.001–1.033) [16]. However, there are also studies reporting different results. A recent study including more than 2.7 million hospitalization cases in Brazil found that higher temperature constantly increased the risks of overall kidney diseases (*RR* = 1.009, 95% CI 1.008–1.010), but reported an inverse association between increasing temperature and CKD admissions (*RR* = 0.998, 95% CI 0.996–1.000) [9]. Another study among about 1.3 million hospital admission cases because of genitourinary system diseases in South Korea did not find statistically significant associations between extreme temperature and CKD [11]. These inconsistent evidences might be attributed to different temperature distribution, spectrum of CKD, age structure or medical system of the study region. Moreover, the distinct medical system and socioeconomic status might also be the potential factors leading to the difference.

Our study further evaluated the association among different temperature zones in China, and found that the association between heat exposure and risk of CKD was stronger in cities in the sub-tropical zone, which is characterized by hotter climate. One possible explanation is that rising temperature increases the risks of recurrent heat exposure, resulting in dehydration and potential impairment in kidney function [4, 10]. Accordingly, exposure to high temperature over a relatively short period may induce the risks of acute progression of CKD. The results of lag patterns showed that the effect of high temperature on hospitalizations for CKD was immediate and strongest at the lag 0 d, which was in line with the previous study [9]. The rapid effect further stressed the necessity of protection strategies for susceptible population in hot season.

Compared with previous studies, our study provided more systematic evidence on the association between ambient temperature and cause-specific CKD [9, 11]. Higher ambient temperature was associated with increased risks of hospitalizations for GN and ON, and ON had the greatest difference with other etiologies. Although this association for patients with DKD and HTN was not significant, our results have showed that heatwaves had significant adverse effects on these etiologies. Our recent study found that there were significantly positive associations between the daily mean temperature and ON hospital admissions in Wuhan, China, which was consistent with this study [47]. There were also some previous studies illustrating that urolithiasis, one of the most causes of ON, was associated with high temperature exposure [48–50]. Epidemiologic evidence is rare regarding the association between heat exposure and hospitalizations for GN, DKD, and HTN. A recent study in Vietnam found that daily mean temperature was associated with risk of hospitalizations for glomerular diseases (odds ratio for every 1 °C increase in daily mean temperature = 1.07, 95% CI 0.99–1.16) through admission data from 14 province-level hospitals, which was in line with our results [14]. Another study conducted in four Philippine cities from 2006 to 2011 found higher risks of diabetes mortality in extreme high temperatures [51]. Unlike the inverse association between short-term high temperature and blood pressure [52], our study found hot exposure was positively associated with hypertension-related CKD. Therefore, more large-sample studies from different regions are still needed to illustrate the relationship between temperature and different etiologies of CKD.

According to our results, younger people may be more susceptible to the health effects of temperature on CKD. Similar findings have been observed in the previous study [21]. One possible explanation is that younger people may spend more time outdoors than older people under the same condition [53], which may increase their exposure to high temperatures.

Furthermore, our study indicated a significant added effect of heatwave in the association between high temperature and hospitalizations for CKD, and results showed that as an extreme heat exposure, heatwaves affect a wider range of populations than general heat. However, previous studies suggested it was not universal that heatwave had added effects above the effect of single days of high temperature on risk of mortality [27] and all-cause hospitalizations [28]. One study among the elderly in the United States observed a significant added heatwave effect for renal admissions but not for cardiovascular diseases, which was consistent with our findings [54]. The result suggested that in addition to high temperature, the duration of extreme temperature might have a more significant effect on kidney diseases than other diseases, which required the attention of vulnerable populations.

The underlying mechanisms for the association of heat exposure with hospitalizations for CKD have not been insufficiently elucidated. High temperature can induce dehydration and blood hyperosmolality, and both of them are considered to result in the development and progression of CKD [10]. Patients with CKD are vulnerable to kidney injury under adverse conditions [55], which can be triggered through different pathways including prerenal, intrinsic renal and postrenal etiologies [56]. For the mechanism of prerenal injury caused by high temperature, one of the possible explanations may be that hypovolemia in hot conditions can result in renal hypoperfusion [57]. And for the intrinsic renal injury, the most common are glomerulus or tubule damage [58]. Experimental studies have proved that heat exposure could lead to renal inflammation and tubular injury in experimental animals [59–61]. Heat exposure can induce dehydration, and then stimulate the secretion of vasopressin, which may result in tubular and glomerular injury [59]. Postrenal injury is due to extrarenal obstruction of urinary flow [58], and our study found that hospitalizations for ON were most strongly affected by ambient temperature. ON is caused by urinary tract obstruction and one of the most common causes of acute urinary tract obstruction is kidney stones. High temperature could contribute to kidney stone formation through heat-induced sweating and concentration of relatively insoluble salts [62], thereby promoting the progression of ON. Nevertheless, it is still necessary to further study the biological mechanisms for the short-term temperature-CKD relationship.

This national study in China investigated the associations between heat exposure and the risks of cause-specific CKD. Our study has a wide geographical distribution covering 261 major cities and over 0.7 million CKD cases, so it provides relatively robust epidemiological evidence on this topic. Moreover, uniform statistical methods were used in this study, and multiple sensitivity analyses were performed to test the robustness of the findings. However, several limitations of this study should be mentioned. First, due to the administrative regulations on individual privacy protection, the residential addresses of the CKD cases were not accessible in this study. Accordingly, we adopted the time series ecological design, which can appropriately evaluate the exposure–response associations at the population level on a day-to-day basis and has been widely used in previous studies [3, 6]. Nevertheless, this study design may be subject to the ecological fallacy and exposure misclassification, and therefore may underestimate the exposure–response associations [63, 64]. Also, since the individual-level confounders were not able to be controlled in this city-level study, the estimated hospitalization risks should be interpreted cautiously. Second, only tertiary hospitals were included in our analyses, which might have led to population selection bias, and the results should be interpreted cautiously when generalized to the total population in China. Third, the association may not necessarily imply causality, which is a common limitation of the observational design. Accordingly, further experimental studies are still warranted to examine our findings. Finally, data on estimated glomerular filtration rate or proteinuria were not available for all patient records in our database. The diagnosis of CKD was based on the ICD-10 coding with relatively low sensitivity and high specificity, which may lead to potential missing of the CKD hospitalization cases [25]. Nevertheless, the high specificity (97.8%) of the ICD-10 coding may effectively improve the robustness of the findings [25].

## Conclusions

In conclusion, our findings suggested that short-term exposure to high temperatures may increase the risks of hospitalizations for CKD in China. People in regions with hotter climates, younger patients and those with ON may be more susceptible. Exposure to heatwaves had significant additional effect on CKD hospitalizations. Under the global background of climate change, our findings evaluated the current heat-related disease burden for CKD in China and provided a novel insight into the effect of high temperature on cause-specific CKD based on empirical evidence from a large-scale population study. Furthermore, our findings implicated the necessity of guided protection strategies against the health effects of high temperature, particularly for the most vulnerable populations.

### Supplementary Information

**Additional file 1: Table S1** ICD-10 codes of various CKD etiologies. **Table S2** Sensitivity analysis results on different model parameters. **Fig. S1** National averaged cumulative exposure-response curve of hospitalizations for CKD associated with ambient temperature in hot season. **Fig. S2** Association between every 1 °C increase in daily mean temperature and hospitalizations for CKD at lag 0–7 d by sex (**a**), age (**b**), and etiology of CKD (**c**). **Fig. S3** Exposure-response curves for different knot placements of temperature-response dimension in the cross-basis function.

## Data Availability

The data that support the findings of this study are available from the Bureau of Medical Administration and Medical Service Supervision, National Health Commission of China, but restrictions apply to the availability of these data, which were used under licence for the current study and so are not publicly available. Data are however available from the authors upon reasonable request and with permission of the Bureau of Medical Administration and Medical Service Supervision, National Health Commission of China.

## Abbreviations

AC: Attributable case
AF: Attributable fraction
CKD: Chronic kidney disease
CI: Confidence interval
df: Degree of freedom
DOY: Day of year
DKD: Diabetic kidney disease
GCV: Generalized Cross Validation
GN: Glomerulonephritis
HQMS: Hospital Quality Monitoring System
HTN: Hypertensive nephropathy
ICD: International Classification of Diseases
ON: Obstructive nephropathy
RR: Relative risk
