# Supplementary material for: Heat exposure and hospitalizations for chronic kidney disease in China: a nationwide time series study in 261 major Chinese cities
Source: Mil Med Res. 2023 Sep 5;10:41. doi: 10.1186/s40779-023-00478-4 (PMC10478241; doi:10.1186/s40779-023-00478-4)
Supplement: Supplementary file 1 — Additional file 1: Table S1 ICD-10 codes of various CKD etiologies. Table S2 Sensitivity analysis results on different model parameters. Fig. S1 National averaged cumulative exposure-response curve of hospitalizations for CKD associated with ambient temperature in hot season. Fig. S2 Association between every 1 °C increase in daily mean temperature and hospitalizations for CKD at lag 0–7 d by sex (a), age (b), and etiology of CKD (c). Fig. S3 Exposure-response curves for different knot placements of temperature-response dimension in the cross-basis function. [file 40779_2023_478_MOESM1_ESM.pdf]

Table S1 ICD-10 codes of various CKD etiologies

Etiology of CKD	All editions[a]	China edition	Beijing edition	Clinic edition
Diabetes mellitus				
Type 1 diabetes mellitus with renal complications	E10.2 + N08.3			
Type 2 diabetes mellitus with renal complications	E11.2 + N08.3			
Unspecified diabetes mellitus with renal complications	E14.2			
Malnutrition-related diabetes mellitus with renal complications		E12.200 + N08.3		E12.200
Other specified diabetes mellitus with renal complications		E13.2		E13.200
Hypertensive diseases				
Hypertensive renal disease with renal failure	I12			
Hypertensive heart and renal disease with (congestive) heart failure	I13			
Pregnancy with hypertensive heart and renal disease	O10.301			
Pregnancy with essential hypertension and proteinuria	O11.x01			
Pre-existing hypertensive renal disease during pregnancy, childbirth and puerperium		O10.200		O10.200
Pregnancy with hypertensive renal disease		O10.201		O10.201
Pre-existing hypertensive heart and renal disease during pregnancy, childbirth and puerperium		O10.300		O10.300
Pre-existing hypertension with proteinuria		O11.x00		O11.x00
Glomerular diseases				
Recurrent and persistent hematuria	N02			
Chronic nephritic syndrome	N03			
Nephrotic syndrome	N04			
Unspecified nephritic syndrome	N05			
Isolated proteinuria with specified morphological lesion	N06			
Persistent proteinuria, unspecified	N39.1			
Obstructive nephropathy				
Hydronephrosis with ureteropelvic junction obstruction	N13.0			
Hydronephrosis with ureteral stricture, not elsewhere classified	N13.1			
Hydronephrosis with renal and ureteral calculous obstruction		N13.2	N13.2	N13.200
Other obstructive nephropathy		N13.8	N13.8	N13.801

[a] This value is given when the codes of the three versions are consistent. *ICD* International Classification of Diseases, *CKD* chronic kidney disease

Table S2 Sensitivity analysis results on different model parameters

Parameter	Ambient high temperature [*RR* (95% CI)]	Heatwave [Added *RR* (95% CI)]
max_lag (d)		
4	1.009 (1.006 – 1.012)	1.058 (1.023 – 1.095)
5	1.008 (1.004 – 1.012)	1.075 (1.036 – 1.115)
6	1.008 (1.004 – 1.012)	1.099 (1.056 – 1.143)
7	1.008 (1.003 – 1.012)	1.116 (1.069 – 1.166)
8	1.008 (1.004 – 1.013)	1.128 (1.076 – 1.182)
9	1.011 (1.006 – 1.016)	1.143 (1.087 – 1.202)
df for humidity		
2	1.009 (1.004 – 1.013)	1.100 (1.065 – 1.157)
3	1.008 (1.003 – 1.012)	1.116 (1.069 – 1.166)
4	1.008 (1.003 – 1.012)	1.124 (1.075 – 1.175)
5	1.008 (1.003 – 1.012)	1.113 (1.065 – 1.164)
df for day of year		
2	1.010 (1.006 – 1.015)	1.094 (1.049 – 1.141)
3	1.008 (1.003 – 1.012)	1.116 (1.069 – 1.166)
4	1.010 (1.005 – 1.014)	1.080 (1.033 – 1.129)
5	1.010 (1.005 – 1.015)	1.085 (1.037 – 1.135)
df for calendar day		
2/year	1.003 (0.999 – 1.007)	1.062 (1.019 – 1.107)
3/year	1.008 (1.003 – 1.012)	1.116 (1.069 – 1.166)
4/year	1.013 (1.008 – 1.017)	1.064 (1.014 – 1.116)
5/year	1.010 (1.005 – 1.015)	1.093 (1.042 – 1.146)
Adjusted air pollutants		
PM_{10}+ O_3	1.008 (1.003 – 1.012)	1.116 (1.069 – 1.166)
$PM_{2.5}$ + NO_2	1.007 (1.003 – 1.011)	1.122 (1.074 – 1.172)
PM_{10} + NO_2	1.007 (1.003 – 1.011)	1.117 (1.069 – 1.166)
$PM_{2.5}$ + O_3	1.008 (1.003 – 1.012)	1.120 (1.073 – 1.170)

CI confidence interval, *df* degree of freedom, PM_{10} particles with an aerodynamic diameter of 10 micrometers or less, $PM_{2.5}$ particles with an aerodynamic diameter of 2.5 micrometers or less, NO_2 nitrogen dioxide, *RR* relative risk

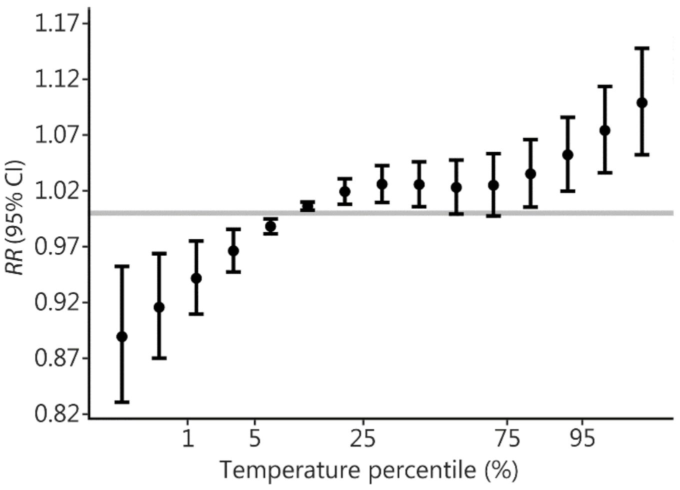

Fig. S1 National averaged cumulative exposure-response curve of hospitalizations for CKD associated with ambient temperature in hot season. The gray horizontal line indicates *RR* = 1. CKD chronic kidney disease, *RR* relative risk

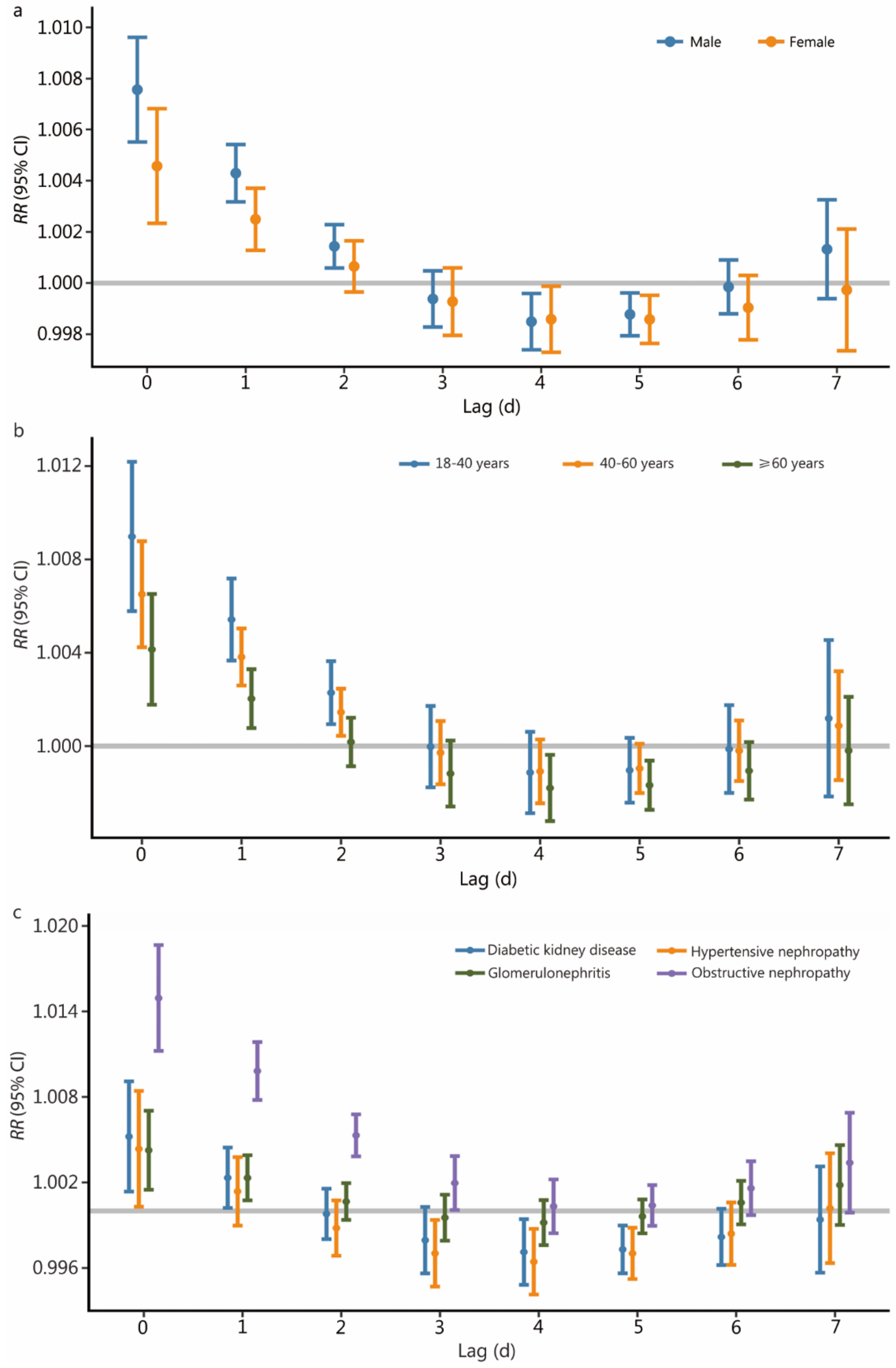

Fig. S2 Association between every 1 °C increase in daily mean temperature and hospitalizations for CKD at lag 0 – 7 d by sex (**a**), age (**b**), and etiology of CKD (**c**). The gray horizontal line indicates *RR* = 1. CKD chronic kidney disease, *RR* relative risk

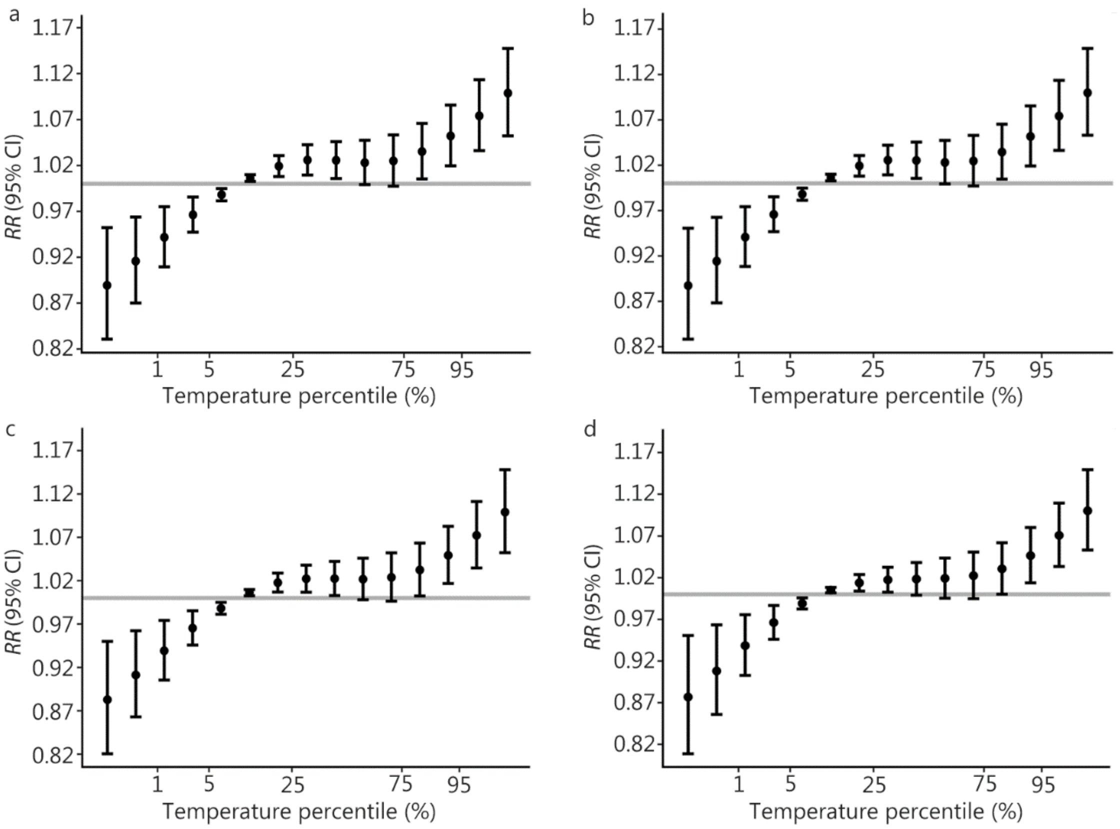

Fig. S3 Exposure-response curves for different knot placements of temperature-response dimension in the cross-basis function. **a** Equal spaced knots. **b** Knots at 30th and 70th percentile. **c** Knots at 20th and 80th percentile. **d** Knots at 10th and 90th percentile. The gray horizontal line indicates *RR* = 1. *RR* relative risk
